# ChatGPT can yield valuable responses in the context of orthopaedic trauma surgery

**DOI:** 10.1002/jeo2.12047

**Published:** 2024-06-17

**Authors:** Janina Kaarre, Robert Feldt, Bálint Zsidai, Eric Hamrin Senorski, Emilia Möller Rydberg, Olof Wolf, Sebastian Mukka, Michael Möller, Kristian Samuelsson

**Affiliations:** ^1^ Department of Orthopaedics, Institute of Clinical Sciences Sahlgrenska Academy, University of Gothenburg Gothenburg Sweden; ^2^ Sahlgrenska Sports Medicine Center Gothenburg Sweden; ^3^ Department of Orthopaedic Surgery, UPMC Freddie Fu Sports Medicine Center University of Pittsburgh Pittsburgh Pennsylvania USA; ^4^ Department of Computer Science and Engineering Chalmers University of Technology Gothenburg Sweden; ^5^ Department of Health and Rehabilitation, Institute of Neuroscience and Physiology Sahlgrenska Academy Gothenburg Sweden; ^6^ Department of Orthopaedics Sahlgrenska University Hospital Mölndal Sweden; ^7^ Department of Surgical Sciences, Orthopaedics Uppsala University Uppsala Sweden; ^8^ Department of Surgical and Perioperative Sciences (Orthopaedics) Umeå University Umeå Sweden

**Keywords:** AI, artificial intelligence, large language models, LLMs trauma orthopaedics

## Abstract

**Purpose:**

To assess the possibility of using Generative Pretrained Transformer (ChatGPT) specifically in the context of orthopaedic trauma surgery by questions posed to ChatGPT and to evaluate responses (correctness, completeness and adaptiveness) by orthopaedic trauma surgeons.

**Methods:**

ChatGPT (GPT‐4 of 12 May 2023) was asked to address 34 common orthopaedic trauma surgery‐related questions and generate responses suited to three target groups: patient, nonorthopaedic medical doctor and expert orthopaedic surgeon. Three orthopaedic trauma surgeons independently assessed ChatGPT's responses by using a three‐point response scale with a response range between 0 and 2, where a higher number indicates better performance (correctness, completeness and adaptiveness).

**Results:**

A total of 18 (52.9%) of all responses were assessed to be correct (2.0) for the patient target group, while 22 (64.7%) and 24 (70.5%) of the responses were determined to be correct for nonorthopaedic medical doctors and expert orthopaedic surgeons, respectively. Moreover, a total of 18 (52.9%), 25 (73.5%) and 28 (82.4%) of the responses were assessed to be complete (2.0) for patients, nonorthopaedic medical doctors and expert orthopaedic surgeons, respectively. The average adaptiveness was 1.93, 1.95 and 1.97 for patients, nonorthopaedic medical doctors and expert orthopaedic surgeons, respectively.

**Conclusion:**

The study results indicate that ChatGPT can yield valuable and overall correct responses in the context of orthopaedic trauma surgery across different target groups, which encompassed patients, nonorthopaedic medical surgeons and expert orthopaedic surgeons. The average correctness scores, completeness levels and adaptiveness values indicated the ability of ChatGPT to generate overall correct and complete responses adapted to the target group.

**Level of Evidence:**

Not applicable.

**Abbreviations**
AI: artificial intelligence
ChatGPT: Generative Pretrained Transformer
LLMs: large language models
SD: standard deviation
USMLE: US Medical Licensing Examinations

## INTRODUCTION

Orthopaedic trauma surgery encompasses a broad range of patients with various musculoskeletal injuries and conditions. However, the continuously evolving evidence in the domain of orthopaedic surgery presents significant challenges to staying up to date with the most recent knowledge [13]. Recent years witnessed a growing interest in the leverage of technological advances for the improvement of information accessibility and decision‐making in health care [4].

Large language models (LLMs), such as Generative Pretrained Transformer (ChatGPT), have garnered substantial attention that has led to a discussion on their potential applications in medicine [3, 5, 8]. These LLMs have demonstrated capabilities to generate human‐like responses, with proficiency in the assessment of comprehensive medical knowledge, as seen with excellent performance on the US Medical Licensing Examinations [7]. Furthermore, ChatGPT has demonstrated the ability to convey empathy, while also simultaneously providing domain‐specific medical responses to patient questions [1].

Given the great amount of evidence and literature available in orthopaedics, the integration of LLMs into orthopaedic practice and research presents considerable advantages. Successful implementation could facilitate improved access to domain‐specific information, enhance clinical and surgical decision‐making and ultimately improve the efficiency and reliability of patient care. Therefore, the primary purpose of this study was to assess the feasibility of using ChatGPT as a source of information or guidance, specifically in the context of orthopaedic trauma surgery by posing questions to ChatGPT and evaluating the responses by orthopaedic trauma surgeons. Additionally, this study aimed to evaluate the correctness and adaptiveness to respective target groups, as well as assess the completeness of the responses generated by ChatGPT. It was hypothesised that ChatGPT would provide overall good responses with varying levels of correctness, adaptiveness and completeness among the different target groups.

## METHODS

### Data source

Three experienced trauma surgeons, of which all three are qualified clinical and research experts in the field (with at least 5, 10 and 15 years of experience, respectively), were asked to individually construct approximately 10 questions relevant to orthopaedic trauma surgery. A meeting was held after to discuss the questions and exclude any duplicates. These initial questions were then further refined, to ensure simplicity in their syntax and grammar. From the pool of refined questions, a total of 34 carefully selected ones were chosen and incorporated into the present study. These questions can be found in the Supporting Information.

### ChatGPT

This study used ChatGPT, a type of LLM, that leverages a Transformer‐style neural network architecture. ChatGPT is an instance of the LLM family, pretrained on an extensive corpus of data to predict subsequent tokens in a document [10]. It was initially introduced as a research variant in November 2022 [11], however, a more recent iteration powered by the GPT‐4 model was released in March 2023 [9]. This updated model (GPT‐4 of 12 May 12 2023), used in this study, exhibits the capability to generate responses with human‐like qualities, and early signs of general intelligence have been observed [2]. While ChatGPT benefits from its extensive pretraining on a diverse data set, to encompass a wide range of resources and enabling the provision of comprehensive information across various topics, it is not without limitations such as including occasional occurrence of ‘hallucinations’, which may subsequently affect the correctness of the responses [9].

### Prompting and data collection

The efficacy of LLMs like ChatGPT has been identified to be significantly influenced by the methods of prompting employed. Thus, in this study, we followed the principles of ‘Prompt Engineering’, a specialised field that offers valuable guidance to construct effective prompts [6, 12]. Accordingly, a prompt was carefully designed to elicit optimal responses from ChatGPT. ChatGPT was further instructed to assume the role of an expert orthopaedic surgeon and provide answers based on the most recent research findings and best practices in the field. Detailed instructions were provided to define the target audience (patient, nonorthopaedic medical doctor, expert orthopaedic surgeon), specifying their expected knowledge level and comprehensive guidelines were outlined for the desired format of the responses (the specific prompts are presented below). To ensure the feasibility of the assessment process and mitigate potential challenges, response lengths were restricted. By encouraging succinct responses, the model was prompted to provide more pertinent information. However, considering the target audience of medical doctors who frequently employ precise terminology and concepts, slightly longer responses (up to 7 sentences instead of 5) were permitted. ChatGPT was used in zero‐shot mode, meaning no specific examples of questions or expected answers were provided. This mode of operation offers a more challenging yet realistic approach compared to other benchmarks that utilise multiple‐choice or few‐shot settings [7].

To counterbalance the influence of context and order, the sequence of questions was randomised for all three target groups. The same randomised order was utilised for all three target groups (patient, nonorthopaedic medical doctor, expert orthopaedic surgeon). Following each initial prompt and response, the response was copied and the subsequent question was presented following the respective prompt.

Upon completion of response collection, an online questionnaire (Google Sheets) was created for each target group, presenting the questions and corresponding responses. This online questionnaire was further used by the assessors to evaluate the correctness, completeness and adaptiveness of the responses to the target group. The assessors were also given the opportunity to provide additional comments to clarify their assessments. Before the assessment, a meeting was held where detailed instructions were provided, to illustrate how to assess the various criteria. The assessors were provided the opportunity to discuss and evaluate some example questions followed by a discussion on whether they based their evaluation on their clinical knowledge or/and current existing literature. Furthermore, each assessor received the instructions and questionnaire links before responses were evaluated. The evaluation of all three assessors was extracted and summarised (Supporting Information).

### Specific prompts

#### Target group ‘patient’

Your task is to answer questions about fractures and treatment options.

I will write questions to you, and you will answer based on the latest, state‐of‐the‐art knowledge and on current established standards for treatment. I want you to only reply with your brief answer, nothing else. Your main goal is that your answers are correct (in line with latest knowledge), as complete as possible (covers the key information) and adapted to the target group.

The target group is a patient that is an adult that has no specific medical education, training or experience.

Your answers need to be understandable and rather brief, preferably two to three sentences and not longer than five sentences. Please do not use overly complex language or wording: the goal is to be clear, direct and understandable.

You cannot assume the patient has deep knowledge of anatomy or physiology, nor about the jargon or specific terms of the field, but you can assume that the patient has a basic understanding of the human body and its functions.

#### Target group ‘nonorthopaedic medical doctor’

Your task is to answer questions about fractures and treatment options.

I will write questions to you, and you will answer based on the latest, state‐of‐the‐art knowledge and on current established standards for treatment. I want you to only reply with your brief answer, nothing else. Your main goal is that your answers are correct (in line with latest knowledge), as complete as possible (covers the key information) and adapted to the target group.

The target group is a medical doctor that has knowledge of anatomy and physiology and a basic understanding of surgical procedures but has no deeper knowledge about surgery nor about the specific treatment options and their relative merits.

Your answers need to be precise but rather brief, preferably two to three sentences and not longer than seven sentences. You can use complex language and wording: the goal is to be precise, give expert advice and provide a broad sense of multiple treatment options. Your answers should be as complete as possible and not leave out any of the important factors.

You can assume the medical doctor has knowledge of anatomy and physiology and a basic understanding of surgical procedures but has no deeper knowledge about (orthopaedic) surgery nor about the specific treatment options and their relative merits.

#### Target group ‘expert orthopaedic surgeon’

Your task is to answer questions about fractures and treatment options.

I will write questions to you, and you will answer based on the latest, state‐of‐the‐art knowledge and on current established golden standards for treatment. I want you to only reply with your brief answer, nothing else. Your main goal is that your answers are correct (in line with latest knowledge), as complete as possible (covers the key information) and adapted to the target group.

The target group is an expert orthopaedic surgeon that has deep knowledge of anatomy and physiology, as well as a deep understanding of surgical procedures and the specific treatment options and their relative merits.

Your answers need to be precise but rather brief, preferably two to three sentences and not longer than seven sentences. You can use complex language and wording: the goal is to be precise, give expert advice and provide a broad sense of multiple treatment options. Your answers should be as complete as possible and not leave out any of the important factors.

You can assume the expert orthopaedic surgeon has knowledge of anatomy and physiology, as well as a deep understanding of surgical procedures and the specific treatment options and their relative merits.

### Assessment

Independent review and assessment of the responses provided by ChatGPT were carried out by three assessors and orthopaedic trauma surgeons, of which all three are qualified clinical and research experts in the field. The correctness (indicating how correct the response was) was graded using a scale of 0 (incorrect), 1 (partially correct) and 2 (correct). Similarly, the completeness of the responses (reflecting how complete the response was, that is, if it included all the necessary parts) was evaluated on a scale of 0 (incomplete), 1 (partially complete) and 2 (complete). Furthermore, the adaptiveness of the responses to the target group (evaluating how well the response was adapted to the target group, using such terminology) was assessed on a scale of 0 (not adapted), 1 (somewhat adapted) and 2 (well adapted).

### Statistical analysis

The average score ± standard deviation and proportions for each of the three criteria (correctness, completeness and adaptiveness) were calculated. Statistical analysis was performed using custom scripts developed in the Julia programming language, version 1.8.5, specifically designed for mathematical computations.

### Source of funding

No funding was received to conduct this study.

## RESULTS

### Patient as target group

The average correctness of responses provided by ChatGPT was determined to be 1.80 ± 0.2 for patients (Table 1), while the level of completeness and adaptiveness were assessed to be 1.79 ± 0.3 and 1.93 ± 0.1, respectively. A total of 18 (52.9%) of all responses were assessed to be correct (2.0), while 18 (52.9%) and 27 (79.4%) of the responses were assessed to be complete (2.0) and well‐adapted (2.0) to the target group, respectively (Table 2).

**Table 1:** The average correctness, completeness and adaptiveness of ChatGPT's responses.

Variable	Patient	Nonorthopaedic medical doctor	Expert orthopaedic surgeon
Correctness (mean ± SD)	1.80 ± 0.2	1.85 ± 0.2	1.88 ± 0.2
Completeness (mean ± SD)	1.79 ± 0.3	1.9 ± 0.2	1.9 ± 0.2
Adaptiveness (mean ± SD)	1.93 ± 0.1	1.95 ± 0.1	1.97 ± 0.1

Abbreviations: ChatGPT, Generative Pretrained Transformer; SD, standard deviation.

**Table 2:** Breakdown of correctness, completeness and adaptiveness for each target group (patient, nonorthopaedic medical doctor and expert orthopaedic surgeon) assessed by the orthopaedic trauma surgeons.

Variable	Patient	Nonorthopaedic medical doctor	Expert orthopaedic surgeon
Correctness, *n* (%)			
2.00	18 (52.9)	22 (64.7)	24 (70.6)
1.67	11 (32.4)	10 (29.4)	8 (23.5)
1.33	5 (14.7)	1 (2.9)	2 (5.9)
1.00	0 (0.0)	1 (2.9)	0 (0.0)
0.00	0 (0.0)	0 (0.0)	0 (0.0)
Completeness, *n* (%)			
2.00	18 (52.9)	25 (73.5)	28 (82.4)
1.67	11 (32.4)	8 (23.5)	3 (8.8)
1.33	4 (11.8)	1 (2.9)	3 (8.8)
1.00	1 (2.9)	0 (0.0)	0 (0.0)
0 (0.0)	0 (0.0)	0 (0.0)	0 (0.0)
Adaptiveness, *n* (%)			
2.0	27 (79.4)	29 (85.3)	31 (91.2)
1.67	7 (20.6)	5 (14.7)	3 (8.8)
1.33	0 (0.0)	0 (0.0)	0 (0.0)
1.00	0 (0.0)	0 (0.0)	0 (0.0)
0.00	0 (0.0)	0 (0.0)	0 (0.0)

*Note*: The correctness was graded using a scale of 0 (incorrect), 1 (partially correct) and 2 (correct). The completeness of the responses was evaluated on a scale of 0 (incomplete), 1 (partially complete) and 2 (complete), while the adaptiveness of the responses to the target group was assessed on a scale of 0 (not adapted), 1 (somewhat adapted) and 2 (well adapted). This table represents average proportions of fully correct, complete and well‐adapted responses for the three target groups. The scores (2.00, 1.67, 1.33 and 1.00) were calculated based on the scores provided by the assessors.

### Nonorthopaedic medical doctor as target group

ChatGPT demonstrated an average correctness of 1.85 ± 0.2 for medical nonorthopaedic surgeons. The level of completeness and adaptiveness were assessed to be 1.90 ± 0.2 and 1.95 ± 0.1, respectively. Overall, all the scores were shown to be higher for responses provided to medical non‐orthopaedic surgeons compared to responses provided to patients. Among the responses provided by ChatGPT, a total of 22 (64.7%) were determined to be correct (Table 2). Furthermore, 25 (73.5%) and 29 (85.3%) of ChatGPT's responses were assessed to be complete and well‐adapted, respectively.

### Expert orthopaedic surgeon as target group

ChatGPT delivered responses to expert orthopaedic surgeons with an average correctness score of 1.88 ± 0.2. The assessment also revealed a level of completeness at 1.91 ± 0.2 and adaptiveness at 1.97 ± 0.1 (Table 2). Furthermore, a total of 24 (70.5%), 28 (82.4%) and 31 (91.2%) of the responses were determined to be correct, complete and well‐adapted, respectively.

## DISCUSSION

The main finding of the study was that ChatGPT could yield overall good responses in the context of orthopaedic trauma surgery across different target groups, including patients, nonorthopaedic medical doctors and expert orthopaedic surgeons. However, ChatGPT demonstrated variation in correctness, completeness and adaptiveness in its responses among the three target groups. Thus, this variability may be important to consider when contemplating the implementation of LLMs in the field of orthopaedics, as there may be differences in ChatGPT's responses based on the knowledge levels of the individuals posing the questions.

In terms of responses provided to the patient target group, ChatGPT achieved an average correctness score of 1.8 out of 2.0, with corresponding levels of completeness and adaptiveness. These findings raise important questions about the clarity and comprehensibility of ChatGPT's responses for patients. While the overall average scores for all three assessment criteria (correctness, completeness, adaptiveness) were good, it is essential to acknowledge that ChatGPT may face limitations in providing information at the patient level. However, since these responses were assessed by orthopaedic trauma surgeons, the assessment may be limited due to the lack of patient assessors. Therefore, future research may consider including patient assessors to secure a more accurate evaluation of the adaptiveness of the responses.

This study revealed that ChatGPT performed better in addressing the specific knowledge needs of nonorthopaedic medical doctors compared to patients. However, the responses of ChatGPT provided to expert orthopaedic surgeons displayed the highest performance compared to non‐orthopaedic medical doctors and patients, as indicated by higher average correctness scores (1.88 vs. 1.85 vs. 1.80), completeness levels (1.91 vs. 1.90 vs. 1.79) and adaptiveness values (1.97 vs. 1.95 vs. 1.93). These findings illustrate the potential of LLMs, such as ChatGPT, to serve as valuable tools to support the knowledge acquisition of specialised orthopaedic surgeons. However, further investigation is needed to evaluate the accuracy and reliability of ChatGPT's responses. For instance, it is essential to evaluate whether the responses provided by ChatGPT are based on the current evidence and whether ChatGPT is able to address complex queries, thus expanding our understanding of the possibility of implementing these models in clinical orthopaedic practice. These language models could assist with summarising the current literature, and thereby, help clinicians to stay up to date on the current evidence and research without needing to spend several hours for a literature search. Also, nonexpert medical doctors, as well as patients could easily get access to current evidence and medical information by asking a question from the chatbot.

This study also has several limitations. First, despite being pretrained on a large data set comprising numerous resources, ChatGPT also has limitations. One of the limitations has been described to be the possible occurrence of ‘hallucinations’, which can negatively impact the accuracy of responses, leading to the possibility of incorrect information, thus, in this study, specific prompts were used to decrease this risk [9]. Also, while access to general information from the internet is readily available, ChatGPT has limited access to scientific literature. This limitation may restrict its ability to provide the most updated and evidence‐based responses. The reliability of the responses generated by ChatGPT was not specifically evaluated. As a result, it is possible that the responses could have varied if the same question had been asked repeatedly or if the responses had been presented in a different order. However, future research could address this limitation by exploring the consistency and reliability of ChatGPT's responses. ChatGPT‐4 as of 12 May 2023, was the only type of LLMs used in this study; thus, this study could only evaluate the performance of one type of LLMs in the context of orthopaedic trauma surgery. Future studies could consider using several LLMs and comparing their performance. The three‐point response scale used to evaluate the responses was not standardised, which may have limited the objective assessment and interpretation of the responses. Although efforts were made to mitigate this limitation by providing the same instructions and examples to all assessors, future studies could explore the use of standardised evaluation criteria to enhance objectivity and comparability across assessments done by the assessors. Furthermore, intrarater reliability was not assessed in this study leading to possible bias. Finally, it is possible that this study included interobserver bias, where a possible variation in the knowledge of the current evidence among the assessors may have had an impact on the evaluation of the responses. Thus, in such a case, some of the ChatGPT's responses may have been assessed incorrectly due to knowledge gaps among the assessors. Considering these limitations, the study findings should be interpreted with caution, thus, the limiting factors may have affected the study results.

## CONCLUSION

The study results indicate that ChatGPT can yield overall good responses in the context of orthopaedic trauma surgery across different target groups, which encompassed patients, non‐orthopaedic medical surgeons and expert orthopaedic surgeons. The average correctness scores, completeness levels and adaptiveness values indicated the ability of ChatGPT to generate overall correct and complete responses adapted to the target group. These findings highlight the potential of LLMs, such as ChatGPT, as valuable supplementary tools for the acquisition of medical knowledge, specifically orthopaedic trauma surgery, by the patient, nonorthopaedic medical doctor and expert orthopaedic surgeon.

## AUTHOR CONTRIBUTIONS

All listed authors have contributed substantially to this work: literature search, study design, data analysis and primary manuscript preparation were performed by Janina Kaarre, Robert Feldt, Bálint Zsidai and Eric Hamrin Senorski. Emilia Möller Rydberg, Olof Wolf, Sebastian Mukka, Michael Möller and Kristian Samuelsson assisted with the study design, evaluation and interpretation of the results, as well as editing the manuscript. All authors have read and approved the final manuscript to be submitted and published.

## CONFLICTS OF INTEREST STATEMENT

Robert Feldt is the Chief Technical Officer and founder of a software consultancy company (Accelerandium AB). Eric Hamrin Senorski is an associate editor of the *Journal of Orthopaedic and Sports Physical Therapy* (*JOSPT*). Kristian Samuelsson is a member of the Board of Directors of Getinge AB (publ).

## ETHICS STATEMENT

Not applicable since all the responses provided by ChatGPT were based on fixed computational models, which are pre‐trained on pre‐existing data.

## Supporting information

Supporting information.

Supporting information.

Supporting information.

Supporting information.

## Data Availability

The data set analysed during the current study is available from the corresponding author on reasonable request.
