# Supplementary material for: ChatGPT can yield valuable responses in the context of orthopaedic trauma surgery
Source: J Exp Orthop. 2024 Jun 17;11(3):e12047. doi: 10.1002/jeo2.12047 (PMC11180970; doi:10.1002/jeo2.12047)
Supplement: Supplementary file 1 — Supporting information. [file JEO2-11-e12047-s003.docx]

**QUESTIONS**

1. What is the recommended treatment of a femoral shaft fracture in an adult?
2. Which are the indications for radial head replacement after trauma to the elbow?
3. What is the reoperation rate after undisplaced femoral neck fractures in elderly patients?
4. Is surgical fixation mandatory for humeral shaft fractures?
5. Is there a role for wire fixation in displaced distal radius fractures?
6. Which is the expected reoperation rate with arthroplasty within five years after internal fixation of cervical femur fractures classified as Garden I or II?
7. At which time after surgery is it appropriate to remove syndesmosis screws?
8. Which are the operative treatments available for adult proximal humeral fracture?
9. How can the incidence rate of additional later fractures be lowered after the first fracture in patients with osteoporosis?
10. At which intraarticular step is operative treatment indicated for a tibial plateau fracture?
11. What is the recommended operative treatment of a femoral neck fracture in a 30-year-old patient?
12. In which ankle fracture types are medial tenderness and swelling important signs for treatment choice?
13. In what ways can a distal femur fracture fixation be augmented in case of medial comminution?
14. Which are the three most common complications after reverse shoulder arthroplasty for fracture?
15. Does the risk for non-union after non-surgical treatment for clavicle fractures mean that displaced clavicle fractures should be treated primarily with surgery?
16. In which situation can a spanning plate fixation be an appropriate treatment option after distal radius fracture?
17. When is the use of a long working length in plate fixation for distal femur fractures a useful option?
18. Which bone in the foot has the highest risk for avascular necrosis after fracture?
19. What is a Bosworth fracture?
20. Do syndesmosis screws need to be removed?
21. What is the complication rate in elderly patients treated for a displaced femoral neck fracture with internal fixation?
22. What are the main indications for internal fixation of ankle fractures at the level of the syndesmosis?
23. Do non-steroid anti-inflammatory drugs (NSAIDs) negatively affect healing after tibial shaft fracture?
24. How does outcome for pilon-type fractures of the distal tibia differ depending on time to surgery?
25. Has hip fracture surgery within 24 hours after injury been associated with lower mortality than surgery performed later?
26. Which are the five most likely fracture locations for fractures related to osteoporosis in the elderly?
27. What is the gold standard treatment for adult both bone forearm fractures?
28. What are the reasons for choosing a total hip arthroplasty instead of a hemiarthroplasty when treating patients with a displaced femoral neck hip fracture under the age of 70 years?
29. What are the complications and the rates of complications for operative and non-operative treatment of humeral shaft fractures, respectively?
30. What is the recommended treatment of a displaced patella fracture in an adult?
31. In which bone are atypical fractures found?
32. What is the dislocation rate after hemiarthroplasty for femoral neck fractures through a posterior approach?
33. What is the recommended treatment of a displaced (>2mm) scaphoid fracture in an adult?
34. Which distal femur fractures need a medial plate?
